# Climate and Health

**Published:** 1895-12

**Authors:** 


### Climate and Health. Edited under the direction of Prof. Willis L. Moore, Chief of Weather Bureau, by W. F. R. Phillips, M. D. No. 2. A Summary of Statistics for the five weeks ended August 31st, 1895: Washington Weather Bureau, U. S. Department of Agriculture, 1895.

The first number of this journal has been already noticed in the November number of The Homœopathic Physician at page 532. It is published monthly, and, quoting from the introduction, "the number of weeks' statistics in the different issues will be arranged so that the fifty-two weeks of the year will be contained in the twelve numbers. The endeavor will be made to have each issue appear as soon as practicable after the termination of the last period considered in it. The statistics are compiled by the calendar week, and are arranged in the following order: Text, climatological tables, morbidity, and mortality tables. The weeks follow each other in chronological order. The charts are, for convenience of printing and binding, placed after the text and tables of the last week and arranged in chronological order."

